# Spectrophotometric Concentration Analysis Without Molar Absorption Coefficients by Two-Dimensional-Infrared and Fourier Transform Infrared Spectroscopy

**DOI:** 10.1021/acs.analchem.2c04287

**Published:** 2022-12-14

**Authors:** Paul M. Donaldson

**Affiliations:** Central Laser Facility, RCaH, STFC Rutherford Appleton Laboratory, Harwell Science and Innovation Campus, DidcotOX11 0QX, U.K.

## Abstract

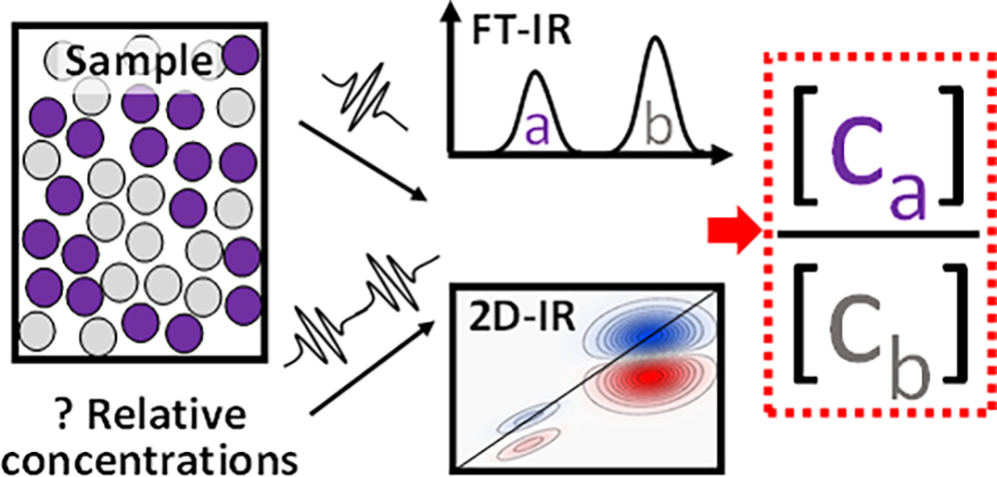

A spectrophotometric method for determining relative concentrations of infrared (IR)-active analytes with unknown concentration and unknown molar absorption coefficient is explored. This type of method may be useful for the characterization of complex/heterogeneous liquids or solids, the study of transient species, and for other scenarios where it might be difficult to gain concentration information by other means. Concentration ratios of two species are obtained from their IR absorption and two-dimensional (2D)-IR diagonal bleach signals using simple ratiometric calculations. A simple calculation framework for deriving concentration ratios from spectral data is developed, extended to IR-pump–probe signals, and applied to the calculation of transition dipole ratios. Corrections to account for the attenuation of the 2D-IR signal caused by population relaxation, spectral overlap, wavelength-dependent pump absorption, inhomogeneous broadening, and laser intensity variations are described. A simple formula for calculating the attenuation of the 2D-IR signal due to sample absorption is deduced and by comparison with 2D-IR signals at varying total sample absorbance found to be quantitatively accurate. 2D-IR and Fourier transform infrared spectroscopy of two carbonyl containing species acetone and *N*-methyl-acetamide dissolved in D_2_O are used to experimentally confirm the validity of the ratiometric calculations. Finally, to address ambiguities over units and scaling of 2D-IR signals, a physical unit of 2D-IR spectral amplitude in mOD/ is proposed.

## Introduction

Spectrophotometric determination of the concentration of analyte molecules by the measurement of absorption of light is a fundamental laboratory technique underpinned by the well-known Beer–Lambert law and the availability of accurate, inexpensive absorption spectrometers spanning the ultraviolet (UV) to the infrared (IR). Quantification of absolute or relative analyte concentrations requires either a molar absorption coefficient to be known, or (equivalently) a spectrophotometric calibration to be performed against a known quantity of the analyte. There are however many chemical research problems where concentration information is required from multi-component samples containing spectroscopically distinct molecular species whose molar absorption coefficients are unknown. The identity of the constituents of these samples may be indeterminate, and their concentrations are difficult to measure by other means, making a spectrophotometric calibration impossible.

An area where obtaining concentrations is challenging is the spectroscopy of transient (“ultrafast”) chemical processes.^1−3^ On photoexcitation of a sample, multiple species may be fleetingly generated and evolve through a series of reaction steps. Obtaining time-dependent concentrations of the intermediates can be key to elucidating reaction mechanisms; however, this is often complicated by the fact that the UV/visible and IR absorption coefficients can be very different for the ground and electronic excited state species generated, requiring further experimental effort and input from theory for their elucidation. The problem of obtaining concentration information from IR spectra may also be encountered in the study of solid catalysts, such as zeolites. Here, the numerous hydroxyl species present give many characteristic IR absorption bands, each corresponding to a hydroxyl group with a unique chemical identity. The hydroxyl stretch (R–OH) vibrational transition strength and frequency vary with R—for zeolites R = Si, Al, and H (H_2_O). Hydrogen bonding causes transition strength to vary by ∼×5 (H_2_O) or more.^4^ A vibration’s molar absorption coefficient is proportional to the square of transition strength, giving a ∼×25 variation in the IR absorption strength from hydrogen bonding alone, accompanied by large variations in IR line shape, which further complicates species concentration analysis. Although IR spectroscopy is extremely common in zeolite studies for ascertaining chemical identity and chemical properties,^5^ it is difficult to use it to accurately quantify relative or absolute concentrations of species of interest, such as Brønsted acid hydroxyls [Si(OH)Al], silanols (SiOH), AlOH, and different types of bound water.^6^

In this paper, a spectrophotometric method for determining relative concentrations of analyte in the absence of absorption coefficients or concentrations is developed and tested. If any one species detectable in an absorption spectrum is of known concentration or absorption coefficient, the other (unknown) species concentrations may then be deduced. The method is built around standard linear absorption measurements such as Fourier transform infrared (FT-IR) spectroscopy and the use of femtosecond two-dimensional (2D)-IR^7^ spectroscopy. It is underpinned by the general principle that IR absorption and 2D spectroscopy signals scale linearly and quadratically with absorption coefficient ε(ν). When the equations for linear and nonlinear absorption are developed side-by-side, 2D-IR signal and IR absorption ratiometric equations can be formulated to eliminate the unknown absorption coefficients and transition dipole strengths, yielding relative concentrations. This principle is explored experimentally in this paper and shown to be accurate to within 20% for two solution phase carbonyl-containing analytes, with the accuracy mostly limited by the stability of the laser used for the 2D-IR experiments, and the means of its characterization.

The FT-IR/2D-IR ratio method presented here was deduced in a similar manner to a related approach presented by the author and Hamm for quantifying IR surface-field-enhanced transition dipole ratios of molecules adsorbed on metal nanoparticle surfaces relative to molecules free in solution, without knowing concentrations of either.^8^ Concurrently, Grechko and Zanni established a similar approach^9^ for determining the variation in amide I transition dipole moment of peptides as a function of secondary structure and later as a function of different types of amyloid fibril formation.^10^ Similar principles were used earlier by Fayer to calculate a transition dipole ratio and equilibrium constant (i.e., a concentration ratio) for characterizing a concentrated salt–water system in a two-state dynamic equilibrium.^11^ Using the properties of linear absorption and nonlinear spectroscopy to calculate transition dipole ratios and concentrations is generalizable, and therefore also appears in other areas of spectroscopy. Building on the idea, Cho et al. published a visible pump–probe method achieving absolute quantification of visible absorbing fluorophore concentrations and their absorption coefficients by also incorporating the measurement of the stimulated emission (SE) photon yield.^12^ Their work mentions a much earlier study by Germann and Rakestraw, demonstrating the use of IR absorption and IR degenerate four wave mixing measurements to determine transition moment ratios and concentrations in high-resolution 1D gas phase spectroscopy.^13^ The present work has much in common with all these earlier studies but provides a deeper practical focus on both 2D-IR spectroscopy application and on the 2D-IR corrections required to obtain accurate results.

The paper is structured as follows: simple expressions describing the strength of linear IR absorption and 2D-IR signal generation are developed to give concentration and transition dipole ratios for two species of unknown molar absorption coefficient/concentration. Similar to previous work,^8,9^ quantities easy to access directly from the experimental data are used—a vibrational band’s peak absorption strength and peak 2D-IR bleach signal intensity. Modification of the equations to calculate ratios using IR pump–probe spectroscopy instead of 2D-IR spectroscopy is also shown to be straightforward. To gain quantitative accuracy, a series of corrections to the 2D-IR signal intensity are described. These account for (1) suppression of the bleach intensity due to overlap with excited-state absorption (ESA) peaks, (2) population and rotational relaxation, (3) line shape broadening mechanisms, (4) nonlinear dependence of 2D-IR signal generation with sample absorption of the pump beam, and (5) spectral variations in pump laser intensity. FT-IR and 2D-IR measurements on mixtures of two organic carbonyl containing species dissolved in D_2_O are presented, and the FT-IR/2D-IR ratio approach to calculating relative concentrations and transition dipole ratios applied. An examination of each correction factor reveals that in these test measurements, the experimental drift in the pump IR laser spectral intensity, the measurement of the IR laser spectral intensity, and the estimation of relaxation effects contributed most to the measurement uncertainty. Finally, attempting to make accurate measurements of 2D-IR signal intensities raises the issue of what the proper choice of 2D-IR signal units should actually be. This issue is not commonly discussed in the literature and therefore it is explored in this paper. An appropriate unit system is proposed.

## Theoretical Background

### FT-IR/2D-IR Expression for Concentration Ratios

A sample’s absorbance *A* of light of frequency ν relates to its molar absorption coefficient ε(ν), concentration [c], and pathlength *L* through the well-known Beer–Lambert law
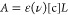
1

The molar absorption coefficient provides the experimental link to the molecular transition dipole moment—the fundamental quantity describing the strength of molecule-field coupling for which many calculations of linear and nonlinear optical phenomena are based on. Under the assumption of a dilute sample, weak illumination, and weak absorption, the molar absorption coefficient is related to the molecular transition dipole strength |μ| via Planck’s constant *h̵*, the speed of light *c*, the vacuum permittivity ε_0_, Avogadro’s number *N*_A_, and refractive index *n*. When the absorbance is in base-ten logarithmic units, the band-integrated molar absorption coefficient relates to a vibrational state transition dipole strength as^14^
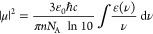
2

Whether the distribution of molecules are isotropic or ordered affects this result by a factor,^8^ but as long as the molecular species being compared in ratiometric calculations have the same orientational distributions, this can be ignored.

In order to develop clear, simple notation for concentration ratio calculations, a number of simplifications are made to eq 2. All constants divide to unity in any FT-IR/2D-IR ratio calculation and are therefore dropped. As a consequence, we may also switch to wavenumber units for frequency (denoted here by ω, as is conventional in 2D-IR spectroscopy). In the IR, the reciprocal frequency dependence of the integral in eq 2 is negligible and dropped. For Lorentzian and Gaussian-shaped absorption lines, the band-integrated molar absorption coefficient term in eq 2 is proportional to the product of the peak amplitude of the absorption coefficient and the full width at half maximum (FWHM) of the band Δω. Combining eqs 1 and 2, a vibrational band’s linear absorbance at peak follows the relation
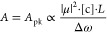
3

Equation 3 is fundamental to this paper. *A*_pk_ and Δω can be simply “read-out” from an IR absorption spectrum as needed. |μ|^2^ and [c] are the unknowns. The pathlength *L* is in-principle easy to measure but typically eliminated in the ratios which follow. Equation 3 applies to homogeneously broadened bands. We will discuss inhomogeneous broadening as a correction in a following section. The next step is to define an equivalent expression to eq 3 applicable to 2D-IR signals. Calculations of 2D-IR signals are generally developed through response function calculations of appropriate Feynman diagrams using a modified density matrix picture, giving a sample ensemble’s time-dependent dipole moment. This is followed by application of classical electrodynamics to compute the emitted signal field.^7^ The key results of such calculations are that under the most common heterodyne detection schemes of three-pulse pump–probe or four-pulse boxcar methods, 2D-IR signals scale as the fourth power of the transition moment, and reciprocal of the homogeneous line width squared.^7^ Signals are linear with concentration and pathlength, although we shall discuss how this assumption is not true at high optical densities. It follows that the analogue of eq 3 describing the peak intensity *S* of a 2D-IR diagonal ground-state bleach-stimulated emission (SE) signal is
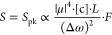
4

For the peak 2D-IR bleach-SE intensity *S*_pk_, the transition moment and homogeneous line width exponents are increased by a factor 2 compared with IR absorption of the same vibrational band (eq 3). The 2D-IR bleach signal size is often attenuated by a number of effects that must be measured or eliminated. These are contained within the additional factor *F* of eq 4 and will be discussed in detail in the next sub-section. Pump–probe bleach-SE signals are similar to eq 4 but scale only as Δω^–1^.

Equations 3 and 4 may be used to develop FT-IR/2D-IR ratio expressions for obtaining transition dipole and concentration information. Similar to previous work,^8,9^ for two homogeneously broadened molecular species a and b, the following ratio of peak 2D-IR bleach-SE signals and FT-IR absorbances relates to the relative transition strength squared as
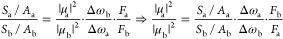
5

The concentrations [c_a_] and [c_b_], as well as the pathlengths *L* cancel in eq 5. Each species’ FT-IR/2D-IR ratio should be measured from the same sample cell for this to hold, though species a and b may be in separate cells.

The aim of this paper is the direct determination of concentrations and elimination of the unknown transition dipole moments. This is achieved using the following ratio, derived by manipulation of eqs 3 and 4 for components a and b at constant pathlength *L*
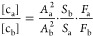
6

Taking the square of the peak IR absorbances *A*_a_ and *A*_b_ achieves cancellation of the transition dipole terms |μ| and line width terms Δω, giving the concentration ratio. If the species are prepared in separate cells of different pathlengths, an extra pathlength factor of *L*_a_/*L*_b_ should be included on the right hand side of eq 6. An experimental test of eq 6 is the focus of the Results section. The modification of eqs 5 and 6 for pump–probe spectroscopy is also discussed in the Results section. To achieve useful accuracy in concentration ratio calculations however, corrections are required to the 2D-IR signal. These are discussed next.

### Corrections to the 2D-IR Signal Measurement

Accurate calculation of a concentration ratio from eq 6 is dependent on the accurate measurement of a sample’s peak absorbance *A*_a_ and *A*_b_ and the peak 2D-IR bleach signals *S*_a_ and *S*_b_ at frequencies ω_a_ and ω_b_. The complicating factor is that unlike linear absorbance values measured from an FT-IR spectrometer in transmission, 2D-IR signals are not absolute quantities—they vary with properties of the sample, instrument, and data collection parameters. This variability is accounted for in this work through the correction factors *F*_a_ and *F*_b_ in eq 6. These corrections factors are broken down into six terms
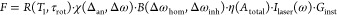
7

**Figure 1:**
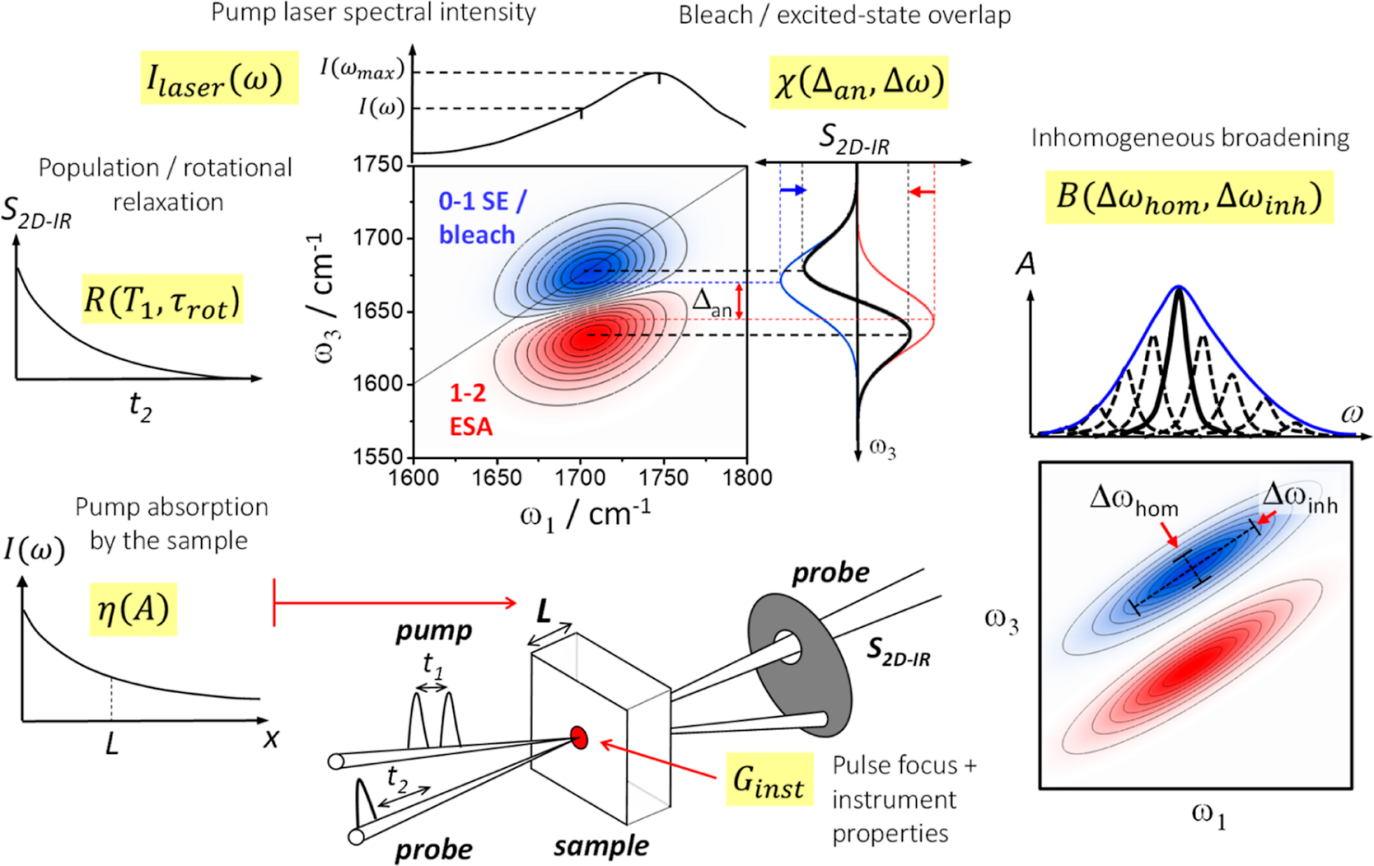
Six factors affecting measured 2D-IR signal intensities (eq 7). These are used in the *F* correction terms of the concentration ratio equation eq 6 and transition dipole eq 5.

The six terms of eq 7 and some of the 2D-IR nomenclature used in this paper are summarized in Figure 1. Each effect is known across the literature of 2D-IR spectroscopy.^7,9,15,16^Eq 7 collectively accounts for them in a manner allowing each to be understood and assessed independently of the others. Equation 7 is heuristic, and line shape terms χ and *B* might be better modeled using global fitting and first-principles 2D-IR line shape functions.^7,17−19^

The first term in eq 7, *R*(*T*_1_,τ_rot_), is the proportion by which the measured 2D signal amplitude is diminished due to population relaxation and rotational dynamics. These effects are species specific and cause the 2D-IR signal *S*_2D-IR_ to decay as a function of the 2D-IR experimental waiting time *t*_2_—the time delay between pump pulse 2 and probe pulse 3 in the three-pulse 2D-IR pump–probe sequence used here. Whether relaxation effects are significant or not depends on the sample’s vibrational relaxation time constant *T*_1_ and the rotational dynamics of the species studied. It may also depend on the choice of 2D-IR waiting time delay *t*_2_ for the measurement and the temporal chirp characteristics of the laser pulses. In some situations, it is not possible to measure accurate bleach-SE amplitudes at *t*_2_ = 0 because instrument response effects perturb the signal. It might also be necessary to collect 2D-IR spectra at increased values of *t*_2_ in order to remove the transient signal of short-lived, broad features from other absorbing species present, such as water,^20^ or to establish a more homogeneous 2D-IR line shape. Rotational dynamics can be eliminated through polarized measurements of the isotropic signal,^7,18^ though this is not necessary for establishing the correction factor in ratiometric calculations. It is shown in Supporting Information Section 2.3 that the experimentally observed 2D-IR or pump–probe signal dependence on waiting time *t*_2_ can be used to establish the correction, regardless of the particular polarization configuration used. One exception arises when comparing isotropic (disordered, e.g., solution) and ordered samples (e.g., crystals, nanoparticle/surface adsorbed molecules). Isotropic transition dipole orientational averaging is assumed in eqs 3 and 4. Should one of the two species being compared have some ordering, the relative variations in orientational averaging terms and rotational dynamics must be accounted for.^8^ For systems with relaxation times comparable to the laser pulse durations, a convolution of the laser pulse envelopes and the observed relaxation should be calculated, as shown in Supporting Information Section 2.3.

### Corrections to the 2D-IR Signal Measurement: Diagonal Peak Overlap

A single vibrational band’s diagonal 2D-IR signal originates from the first three quantum states (0, 1, and 2) of the vibrational mode anharmonic potential. The second term in eq 7, , accounts for the attenuation of the 2D-IR (0–1) diagonal bleach-SE peak caused by overlap with the opposite-signed ESA(1–>2) peak, as shown in Figure 1. The diagonal anharmonic frequency shift Δ_an_ is defined as ω_01_–ω_12_ or 2ω_01_–ω_02_. The correction factor  becomes <1 at the onset of overlap, when Δ_an_ is equal to or less than the homogeneous line width Δω of the bleach-SE and ESA bands. As Δ_an_ → 0, the bleach-SE and ESA peaks overlap completely, and the diagonal 2D-IR signal disappears. For simple 2D-IR peak shapes, determining the correction factor  is straightforward when Δ_an_ and the line width Δω are known or easily measured. Then,  can be estimated by fitting the slice of experimental data along the probe (ω_3_) axis at the pump-axis (ω_1_) band center to a model line shape comprising the sum of two opposite-signed 1D line shape functions of width Δω and separation Δ_an_.  is the ratio of the peak heights between the actual experimental bleach amplitude and the amplitude of the fitted line shape function determined for the bleach, as shown in Figure 1 and Supporting Information Section 2.2. If Δ_an_ is not known, and the separation of bleach-SE and ESA peaks are comparable to Δω, then Δ_an_ should not be read-out from the 2D-IR spectrum by the peak-to-peak separation of bleach-SE and ESA bands—it will be overestimated. Δ_an_ might instead be obtained via measurement of the weak 0–2 overtone transition frequency ω_02_ by FT-IR, by alternative 2D pulse sequences,^21,22^ by 2D-IR line shape simulations,^7,17,18^ or by anharmonic Ab initio vibrational frequency calculations.

### Corrections to the 2D-IR Signal Measurement: Inhomogeneous Broadening

The third term in eq 7, , describes inhomogeneous broadening. The IR absorption and 2D-IR signal equations (eqs 3 and 4) apply to homogeneously broadened molecules. Inhomogeneous broadening can be viewed as “adding” more of the same type of molecules to the sample but absorbing across a range of frequencies, as depicted in Figure 1. The simplest case to correct for is when the inhomogeneous broadening is both static—corresponding to a time invariant distribution of distinct molecular structures, and, when the transition dipole moment is constant with frequency ω (the Condon approximation). To be classified as “static,” the distribution of structures should persist for greater than the population time *T*_1_, ruling out any contributions of dynamical dephasing (spectral diffusion) to the IR and 2D-IR line shape. 2D-IR spectroscopy readily distinguishes homogeneous and static inhomogeneous broadening—the bleach-SE and ESA are inhomogeneously broadened along the diagonal by Δω_inh_ (FWHM) and along the antidiagonal by the homogenous width Δω_hom_ (Figure 1). The on-peak 2D-IR bleach-SE signal intensity is proportional to the concentration of the sub-ensemble of molecules of homogeneous width Δω_hom_ around the center frequency. The remainder of the molecules spanning the inhomogeneous line increase the observed concentration ratio by a factor ∼Δω_inh_/Δω_hom_. This underestimation of concentration can be compensated for using the correction factor
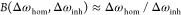
8

2D-IR line shapes can be complicated, and there are several other line broadening scenarios which might be encountered. If the system is undergoing spectral diffusion on a timescale faster than the vibrational relaxation time *T*_1_, the IR line width is affected, and therefore, the correct 2D-IR peak intensity for eqs 5 and 6 is the homogeneous limit (spectrally diffused, long *t*_2_), compensating appropriately for sample relaxation [*R*(*T*_1_,τ_rot_)]. Motional narrowing^7^ may simply be considered as homogeneous broadening, with the 2D-IR anti-diagonal and IR absorption homogeneous line widths equal at short waiting time *t*_2_, as required by eqs 5 and 6. Other effects on IR and 2D-IR line shapes include transition dipole variation with frequency across the band (the non-Condon effect). These might be detected and accounted for by explicitly evaluating the transition dipole ratio formula eq 5 across the inhomogeneous width. Inhomogeneously, broadened hydrogen bonded systems may also show frequency dependence in (i) anharmonic shift, (ii) *T*_1_ relaxation, and (iii) homogeneous line width. At this level of complexity, the *F* correction terms in eq 7 are definitely an over-simplification, and more realistic line shape simulations may be necessary.

### Corrections to the 2D-IR Signal Measurement: Pump Intensity, Attenuation, and Instrument Effects

The expression for the 2D-IR bleach signal (eq 4) appears to allow for unlimited signal generation with increasing concentration, pathlength, and transition strength. In fact, the incident laser beams are attenuated as they propagate across the sample, limiting the maximum signal generated at high optical densities and distorting the 2D-IR line shapes along the pump axis.^16,23^ These effects are accounted for in the transition dipole and concentration ratio correction factor *F* (eq 7) by including a pump attenuation term η, which we develop here using a simple one-dimensional model.

In the three-pulse pump–probe geometry often used for 2D-IR experiments, the 2D-IR signal is generated by and heterodyned by the probe field. As the probe beam propagates across the sample, both the probe field and 2D-IR signal are identically absorbed, so their mutual attenuation by the sample cancels. The effect of the 2D-IR signal and probe absorption on the signal size can therefore be ignored. This is not the case for the pump fields. 2D-IR signal size is pump intensity-dependent and so pump absorption by the sample must be taken into account. Strictly speaking, pump absorption is nonlinear with incident intensity and therefore spot-size dependent. Pump-induced absorption is seldom observed in vibrational 2D-IR spectroscopy at levels higher than 1–2% (0.01–0.02 OD), however, making linear absorption a reasonable approximation. The pump intensity *I*(*x*) is then attenuated as a function of the position *x* across a sample of length *L* as
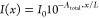
9

*I*_0_ is the incident intensity, and *A*_total_ is the total absorbance of the sample across its pathlength at a given pump frequency
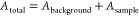
10

*A*_sample_ is the absorbance of the sample component of interest. *A*_background_ is the absorbance from backgrounds and scattering—in other words, optical losses which do not contribute to the 2D-IR signal. For the concentration ratio measurements, the total linear absorption *A*_total_ at the resonant frequency of species a might be different to that of species b, resulting in different levels of pump attenuation and thus different relative signal sizes compared to the ideal case of negligible pump absorption.

The 2D-IR signal is proportional to the concentration of signal-generating sample chromophores (eq 4). Equations 1 and 4 imply that the 2D-IR bleach signal is proportional to the absorption coefficient squared; however, the contribution from the first two pump field interactions is simply proportional to the sample molar absorption coefficient. Along with the pathlength *L*, the product of these three terms is equal to the sample’s absorbance *A*_sample_ (Beer–Lambert law, eq 1). As the pump beam traverses the sample, its contribution to the 2D-IR signal generated at sample position *x* is proportional to its intensity (eq 9). Thus, for the idealized case of weak sample absorption, where the pump absorption is negligible, *S*_2D-IR_^ideal^ ∝ *I*_0_*A*_sample_. When pump attenuation is significant, we proceed as follows. Dividing the sample into slices of thickness Δ*x*, the sample absorbance and laser intensity contribute to the 2D-IR signal generated from each slice as ∼*I*(*x*)·*A*_sample_·Δ*x*/*L*. Incorporating eq 9 for the intensity, we may sum this contribution to the 2D-IR signal strength across the sample length *L* to give the 2D-IR signal dependence on sample absorbance and total absorbance
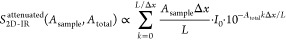
11

The incident pump laser spectral intensity is dealt with through a separate correction factor. Here, we set *I*_0_ to 1. Identifying *k*Δ*x* → *x* and Δ*x* → d*x*, eq 11 integrates across the sample length *L* to give
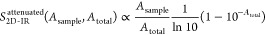
12

**Figure 2:**
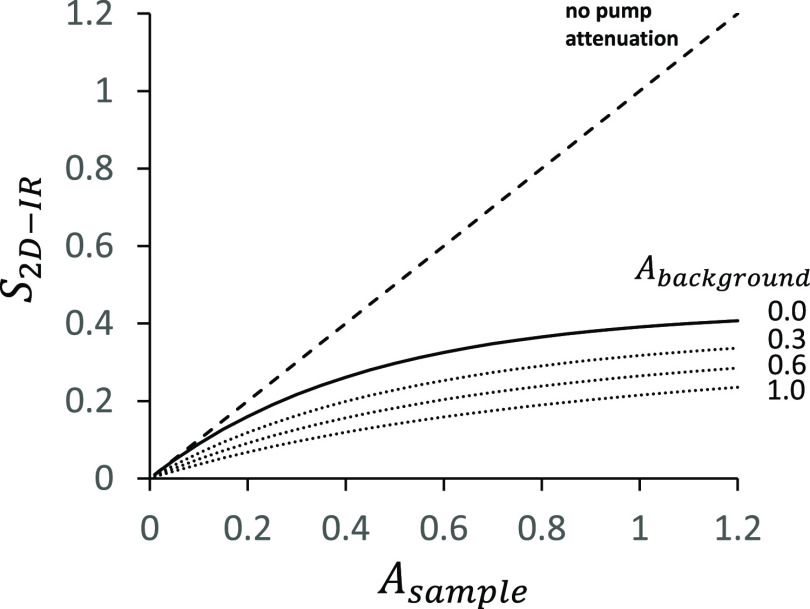
At a given pump frequency, as the absorbance of a sample increases, pump attenuation causes the relative gain in the 2D-IR signal to decrease. A simple model (eq 12) for the 2D-IR signal  is plotted against the sample absorbance *A*_sample_ with increasing contributions of background absorbance, *A*_background_.

Equation 12 is validated in the Experimental Section/Results section (Figure 5). Figure 2 shows how this contribution to the 2D-IR signal scales with sample absorbance. Note that the pathlength of the sample is integrated over and enters in this model only in determining the absorbance of the sample. The nonlinearity in 2D-IR signal generation as a function of absorbance *A*_sample_ > 0.1 is substantial, and additional background absorption further reduces the 2D-IR signal size. For constructing a correction factor to use in eq 7, we require a quantity which describes by how much the 2D-IR signal is reduced compared to the idealized case of no pump attenuation (*S*_2D-IR_^ideal^ ∝ *A*_sample_, diagonal line, Figure 2)
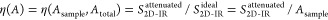
13

For small *A*_sample_ and *A*_background_ = 0,  = 1. Upon increasing either the sample or background absorbance above ∼0.1,  becomes less than one.

The actual values of the pump spectral intensity for each species a and b at ω_a_ and ω_b_ were dropped from eq 12 and from the pump absorption correction factor η(*A*). The pump spectral intensity is a property of the lasers used (rather than the sample) and therefore requires a separate measurement using a spectrometer or interferometry. Similar to previous work,^9^ a correction term in eq 7, *I*_laser_(ω), is used. With conventional optical parametric amplifier (OPA)-based femtosecond IR sources, the pump spectral intensity is typically peaked around a center wavelength. *I*_laser_(ω) is defined here as the ratio of the measured pump intensity at the absorption frequency ω relative to the peak intensity.

2D-IR signal intensities also depend on other instrument factors such as absolute incident pump pulse energy, focal spot size, laser pulse spatial overlap quality, and optical polarization. These are all represented in eq 7 by a general instrument factor *G*_inst_, which must remain constant for ratiometric measurements in order to cancel from eqs 5 and 6. If it is not possible to perform a ratiometric analysis of two species a and b on the same 2D-IR instrument at the same time, a correction to the 2D-IR signal of sample a or b, *G*_inst_^1^/*G*_inst_^2^, should be determined. This could be the ratio of signal sizes of an identically absorbing reference sample for the two separate measurements or instruments used. The correction *I*_laser_(ω) would also need to be redefined appropriately between the measurements. In this work, an FT-IR spectrometer is used to determine IR absorption. For the case of samples that are spatially heterogeneous on a length scale comparable to the 2D-IR and FT-IR beam sizes, it may be necessary to improve on the resulting uncertainty in the IR absorption/2D-IR signal measurements by using the 2D-IR probe beam to measure the IR absorption.^9,10,24,25^ As such measurements suffer from greater noise compared with FT-IR measurements, use of an FT-IR microscope to map the absorption of the sample could be used to increase confidence in the IR absorption measurement.

### Corrections to the 2D-IR Signal Measurement: Units of the Signal Size

It is common in the literature to define 2D-IR signal units as “arbitrary” or not include 2D-IR signal units when presenting 2D-IR data. This is partly explained by the fact that variations in laser and data collection parameters result in 2D-IR signal magnitudes varying in size from one instrument to another (encapsulated by the factor *G*_inst_ defined above). There is another reason however: this is that a definition of units is not so straightforward! As long as 2D-IR signals are measured under repeatable conditions, ratiometric calculations and other applications of 2D-IR spectroscopy do not require the spectra to have defined signal units. Nevertheless, as an analytical technique, a standard set of units for comparing results from different instruments is a requirement. So how to proceed?

For the most common three-pulse pump–probe Fourier transform (FT)-2D-IR geometry, the probe (ω_3_) axis is determined by spectral dispersion of the three-pulse nonlinear signal (also called the third-order response) onto an array detector. The three-pulse signal is both driven by and interfered (heterodyned) with the probe pulse. The 2D-IR spectrum and its dependence on ω_1_ are then determined by FT of three-pulse signal interferograms collected as a function of time delay *t*_1_ for each ω_3_ array detector pixel. The per pixel signal strength is most conveniently expressed in absorbance units using the heterodyning probe beam as a reference. It is important to note that this referencing, and the heterodyne detection itself, has the convenient consequence of normalizing the observed three-pulse nonlinear signal field strength. It also eliminates all dependence of the 2D-IR signal strength on the probe intensity and on the dispersion parameters of the spectrograph resolving the probe. These would otherwise affect the signal units but can instead be dropped from the discussion.

For determining the ω_1_ axis of a 2D-IR spectrum in the frequency domain using the alternative narrowband heterodyned pump–probe approach,^7^ it makes sense to normalize the pump–probe signal by the narrowed pump spectral bandwidth, giving 2D-IR signal units of absorbance/cm^–1^. For the more usual broadband time-domain determination of interest here, the definition for the analytical (continuous) FT implies that the resulting 2D-IR signal units ought also to be absorbance/cm^–1^. The catch is that for FT-2D-IR, the required *t*_1_ coherence time interferograms recorded in a three-pulse pump–probe geometry are discrete and finite—and therefore so is the FT. Discrete 2D-IR acquisition parameters of *t*_1_ step size Δ*t* and finite interferogram spectral resolution (determined by the number of interferogram points *N*) affect the transformed signal size calculated by discrete FT (DFT) algorithms, as will the number of phase cycles *n*_ϕ_ and number of chopped measurements used to isolate the signal from the probe and other backgrounds.

Upon proper normalization, a natural system of signal units in absorbance/cm^–1^ is not forthcoming from a 2D-IR spectrum generated by DFT of discrete interferograms. Under DFT, the square of the signal (power spectrum) gives a 2D-IR spectrum in units of absorbance^2^/cm^–1^, as shown in Supporting Information Section 3. To avoid this issue, an ad hoc normalization, invariant under change of interferogram acquisition parameters was suggested previously by the author—correcting the 2D-IR signal amplitude by scaling it so that the projection (sum of 2D-IR slices) along the pump axis matches the pump–probe signal derived from a chopped “pump-on, pump-off” measurement.^26^ A better, more rigorous normalization independent of pump spectral resolution and *t*_1_ coherence time step size Δ*t* may instead be defined with the 2D-IR signal in units of absorbance/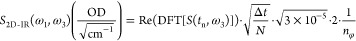
14

This scaling applies to the transform of zero-padded interferograms with step size measured in femtoseconds. It is straightforward to apply after FFT, retains physical (rather than arbitrary) units for the signal amplitude, and is independent of the pump spectral resolution, sampling interval, and phase cycling scheme. The peculiar requirement of the square-root scaling of the frequency appears as a consequence of the discrete FT. This is further discussed and explored in Supporting Information Section 3, and a scaling to match the projected 2D-IR spectrum to the pump–probe spectrum demonstrated.

## Experimental Section

### Samples

The FT-IR/2D-IR concentration ratio calculation eq 6 was tested by preparing 17 solutions of acetone and *N*-methyl-acetamide (NMA) dissolved in 1 mL of D_2_O in the range of 50–150 mM. Calibrated pipettes (Eppendorf) were used for dispensing the NMA and acetone in ∼10 μL volumes. The NMA (melting point 25 °C) and pipette tips were maintained at 40 °C to prevent solidification of NMA during dispensing. Repeatability of preparations of specific concentrations of solution was found to be poor. Volume dispensing was found to be only 10–30% accurate for the small dispensed amounts of volatile, low-viscosity acetone, and the viscous, easily solidified NMA. The three substances were also weighed in their 1 mL container tubes using a precision balance (Ohaus Pioneer Semi-Micro), but as NMA is toxic, the solutions had to be prepared and weighed in a fumehood. Although the specified repeatability (±0.1 mg) of the balance was more than sufficient to achieve good accuracy, operation in a fumehood and the low (4–7 mg) amounts dispensed lowered the repeatability of mass determination to around ±1 mg.

The low preparation accuracy of the test solutions described above was turned into an advantage—the 17 mixtures for FT-IR/2D-IR tests were prepared on-demand with low accuracy, as described above, giving a randomized spread of unknown concentration ratios to examine using the FT-IR/2D-IR ratio method and by the Beer–Lambert method. For the Beer–Lambert characterization, the determination of acetone and NMA molar absorption coefficients in D_2_O was achieved using an independent set of standards prepared with higher accuracy from several higher concentration solutions of NMA and acetone. These were prepared using much larger weighed amounts of solvent and solute (5 mL D_2_O, ∼1 M solute concentration) and then weighed volume dilution.

### Sample Spectroscopy Cells

A pair of commercial compression-sealed spectroscopy cells (Harrick) were used for IR and 2D-IR measurements. These had 2 mm CaF_2_ windows and pathlengths set by 25 μm PTFE spacers. The same cell and solution were used for each sequence of FT-IR and 2D-IR spectroscopy, with each pair of FT-IR and 2D-IR measurements conducted within minutes of the other. Variable pathlength studies were conducted using combinations of 6, 10, 25, 50, and 100 μm PTFE spacers. The spacers were laser cut in-house from sheets of PTFE (Goodfellow). For the 17 low accuracy concentration samples, pathlength variability was found to be ±15% per preparation. For Beer–Lambert concentration determination, this was corrected for by using the strength of the IR absorption band of D_2_O at 1570 cm^–1^.

### Infrared Spectroscopy

A Bruker Tensor FT-IR spectrometer equipped with a DGTS detector was used to determine the FT-IR absorption spectra of the prepared solutions and of pure D_2_O against a background of air. 2 cm^–1^ spectral resolution and 32 scans (10 kHz scan velocity) were used for all measurements with the exception of the determination of the line widths and overtone frequency of acetone, where a 1 cm^–1^ spectral resolution and 260 scan measurement were used.

### 2D-IR Spectroscopy

Practical and technical details can be found in Supporting Information Section 1.

## Results and Discussion

### IR and 2D-IR Spectroscopy of Solutions of NMA and Acetone in D_2_O

**Figure 3:**
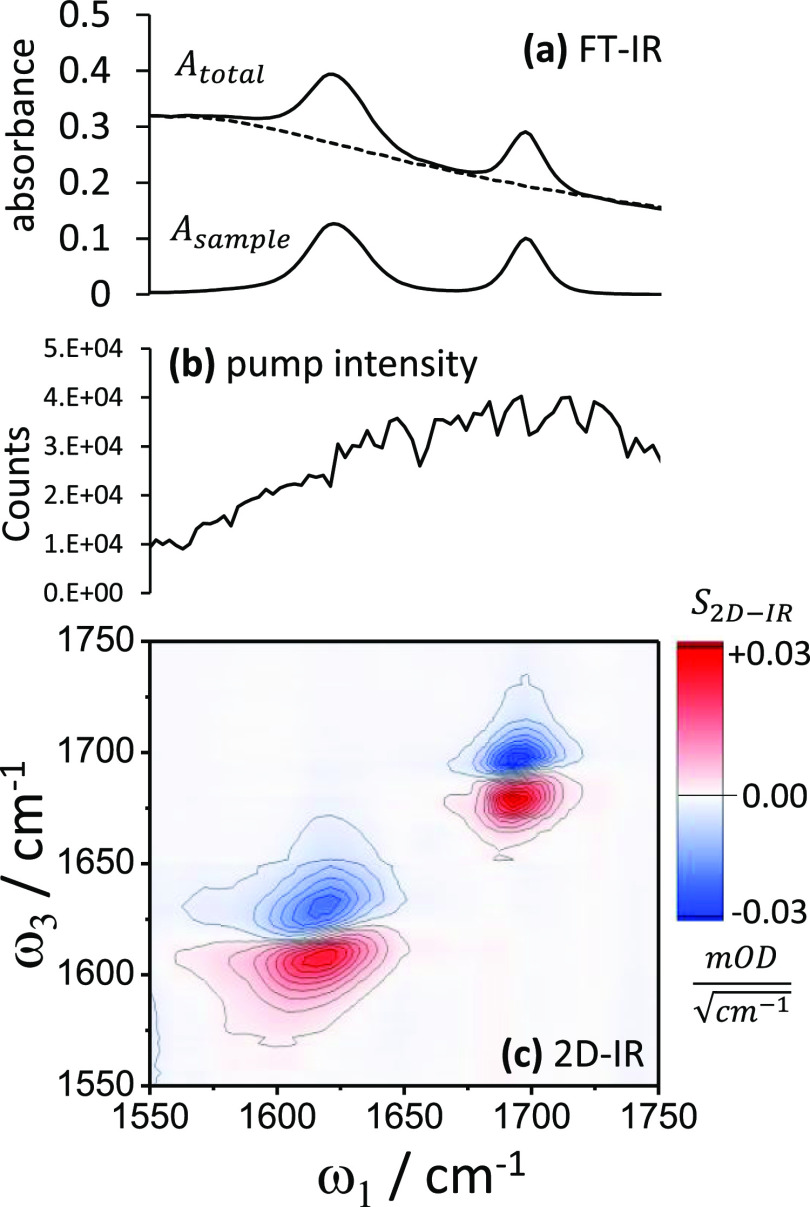
FT-IR (a) and 2D-IR spectrum (c) of a 25 μm pathlength sample of acetone and NMA dissolved in D_2_O (*t*_2_ ≈ 300 fs). For this particular sample, the acetone is 94 mM, and NMA is 70 mM in concentration. The FT-IR spectrum comprises total absorption (*A*_total_), the D_2_O background (*A*_background_, dotted, measured separately), and the background subtracted spectrum (*A*_sample_). Shown center (b) is an intensity spectrum of the pump beam.

In order to test the FT-IR/2D-IR concentration ratio equation (eq 6), a set of solutions of NMA and acetone in D_2_O were prepared over a range concentration ratios, as described in the Experimental Methods Section. The choices of NMA and acetone as test solutes were based on the two types of molecules containing spectrally distinct hydrogen-bonded organic carbonyl stretch modes at 1623 cm^–1^ (NMA) and 1698 cm^–1^ (acetone). Aqueous solutions of NMA have been studied extensively by 2D-IR spectroscopy, so much is known about their spectral properties.^27−31^Figure 3 shows representative IR and 2D-IR spectra of one of the solutions studied. The carbonyl stretch IR bands lie on top of a background absorption from D_2_O (which is due to the latter’s combination band of bend and libration modes). The relaxation of this D_2_O band was too weak/fast to observe with the 2D-IR spectrometer, and therefore, any possible background made minimal contributions to the 2D-IR spectra. Peak bleach-SE signal values were used “as-read” from the intensity (*z*) axis of the 2D-IR spectra. For the FT-IR measurements, on-peak IR absorption values (*A*_sample_) were determined from the total absorbance (*A*_total_) for each measurement by subtracting a separately recorded spectrum of D_2_O, taking into account the variation in background due to pathlength variations. The acetone and NMA carbonyl IR bandwidths Δω were observed to be 16 and 27 cm^–1^ (FWHM, ±0.5 cm^–1^). A pump intensity spectrum recorded prior to commencing measurements 1–17 is shown in Figure 3b.

**Figure 4:**
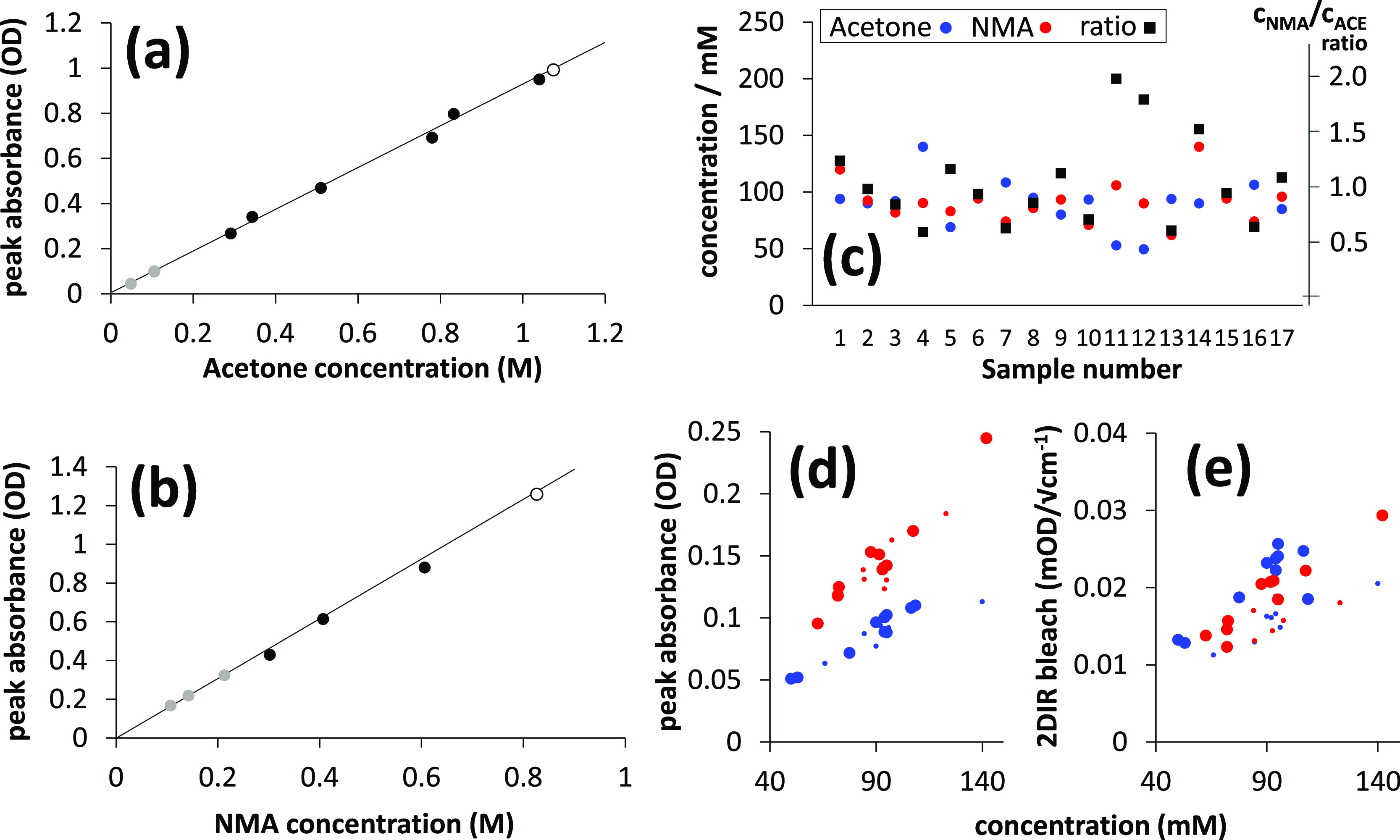
Accurate determination of the molar absorption coefficients of acetone/NMA carbonyl stretch bands by the Beer–Lambert law and application to the characterization of 17 random samples prepared for FT-IR/2D-IR ratiometric analysis. (a,b) Beer–Lambert calibrations, showing acetone and NMA carbonyl peak absorbance *A*_sample_ vs concentration plots (D_2_O, 25 μm pathlength). The colors (white, black, and gray) are three separate mass preparations. Multiple points of the same color are dilutions. (c) Concentrations and concentration ratios of 17 random test samples used for ratiometric analysis, as determined using the Beer–Lambert molar absorption coefficients from (a,b). The 17 test sample carbonyl stretch peak IR absorption *A*_sample_ and 2D-IR bleach-SE magnitudes *S* are shown as a function of the concentration in (d,e). Red points are NMA, and blue points are acetone. Small and large points distinguish two consecutive measurement sessions 1–7 and 8–17.

For Beer–Lambert law analysis, peak molar absorption coefficients of the acetone and NMA carbonyl stretch bands were determined using six solutions prepared accurately at high volume and concentration (∼1 M), then diluted using both calibrated pipetting and weighing. Plots of background subtracted FT-IR peak absorption (*A*_sample_) versus gravimetrically determined concentration are shown for these Beer–Lambert calibration solutions in Figure 4a,b. Up to 1 M, the acetone and NMA carbonyl stretch peak absorption values are linear with the concentration, and the spectral positions/widths of the bands were independent of the concentration over the concentration range studied. Peak absorption coefficients were determined to be 3.69 × 10^4^ (OD) M^–1^ m^–1^ ± 1% (linear regression uncertainty) for acetone and 5.99 × 10^4^ (OD) M^–1^ m^–1^ ± 1% for NMA.

The acetone and NMA concentrations of the 17 random samples were determined using the Beer–Lambert absorption coefficients and the measured absorptions/pathlengths. The range of concentrations and ratios examined are shown in Figure 4c. Across the series, the concentrations and ratios are well randomized. The background subtracted peak IR absorbance (*A*_sample_) values used for the ratio calculations are shown as a function of the concentration for the 17 samples in Figure 4d. The deviation from linearity is due to sample-to-sample pathlength variations. Figure 4e shows the corresponding peak 2D-IR bleach-SE amplitudes for samples 1–17. Measurements 1–7 and 8–17 were conducted on successive days. Over 1–7, the laser performance deteriorated, and adjustments to the regenerative amplifier’s compressor and the probe OPA were necessary prior to commencement of measurements 8–17. As shown in Figure 4e, 2D-IR measurements 1–7 are made distinguishable from measurements 8–17 by using the reduced data-point size. Measurements 1–7 clearly show a lower average 2D signal size.

An important property of the NMA and acetone 2D-IR bleach amplitudes, as shown in Figure 4e, is their approximate parity in strength as a function of the concentration, despite the fact that NMA is the stronger IR absorber. Accounting for the absorption coefficient and line width differences, the NMA 2D-IR signal intensity is clearly underestimated in the raw 2D-IR signal measurements. This is due to the pump laser intensity, diagonal peak overlap (anharmonicity), pump absorption, and relaxation effects, as described in Figure 1 and eq 7. Although a slight tilt is observed in the 2D-IR line shapes at the waiting times used (*t*_2_ = 300 fs), the 2D-IR peaks are close to homogeneous in shape, and the line shape correction factor  was assumed to be ∼1. The other terms of eq 7 for the correction *F* are summarized in Table 1 for both NMA and acetone. The experimental acetone bleach-SE intensity is in total reduced by a factor *F* of around 0.65 (±12%) and the NMA bleach-SE correction by a factor *F* of around 0.26 (±15%). These amount to a significant correction to the determination of the NMA/acetone transition dipole square ratio and concentration ratio: ∼2.5 and ∼0.4, respectively (±20%), (uncertainties calculated from combinations of the individual measurement uncertainties).

**Table 1:** Correction Factors Used for Determining the Transition Dipole Ratios and Concentration Ratios in Figure 7^a^

	laser I (ω)	anharmonicity	pump absorption (Figure 6b)	relaxation *R*(*T*_rel_,τ_rot_)
acetone	1 ± 10%	1	in range of 0.8 ± 2%	∼0.81 ± 5%
NMA	0.82 ± 5%	0.72 ± 4%	in range of 0.7 ± 2%	∼0.62 ± 10%

a The range of pump absorption corrections are shown in Figure 6b.

**Figure 5:**
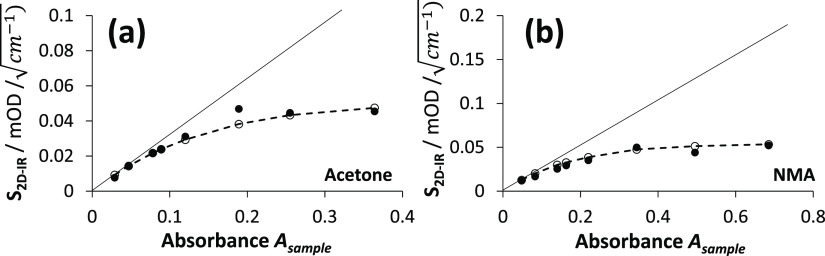
Effects of pump absorption on the 2D-IR bleach-SE signals of the carbonyl stretch of acetone (a) and NMA (b) in D_2_O as a function of the absorption of the sample. A D_2_O absorption background is present for all measurements. The black circles are experimentally observed 2D-IR signal values for samples measured with varying pathlengths, plotted as a function of the FT-IR determined sample absorbance. The spacers used were 6, 10, 16, 25, 35, 50, 75, and 100 μm. The white circles/dotted lines are theoretical 2D-IR signal values computed using eq 12 from the sample and background IR absorbance. The straight line is the idealized case of no pump absorption.

Discussion of determination of the intensity, anharmonicity, and relaxation corrections in Table 1, along with their uncertainties, can be found in Supporting Information Section 2. The pump absorption factor  described by eqs 12 and 13 was derived for the purposes of this paper and therefore requires experimental verification. To this end, measurements of the 2D-IR signal dependence on sample absorption and total absorption *A*_sample_ and *A*_total_ were conducted, the results of which are shown in Figure 5. A solution of NMA and acetone in D_2_O (concentrations ∼95 mM) was examined by FT-IR and 2D-IR over a range of pathlengths from 6 to 100 μm. For these concentrations, the D_2_O background absorbance *A*_background_ was ∼60% of the total absorption for each pathlength. The pump-attenuated 2D-IR signal (eq 12) was calculated using the FT-IR-measured absorbance values for the acetone carbonyl stretch, NMA amide I band, and the D_2_O background. To compare with the experimental data, the eq 12 model 2D-IR signal values were scaled to the value of the experimental 2D-IR bleach-SE signal determined at the lowest sample absorbance (6 μm pathlength, total absorbance <0.13), where the 2D-IR signal is almost linear with sample absorbance. The fit of the calculated signal to the experimental signal points in Figure 5 is excellent, indicating that the approach to calculating the pump absorption correction factor is accurate.

**Figure 6:**
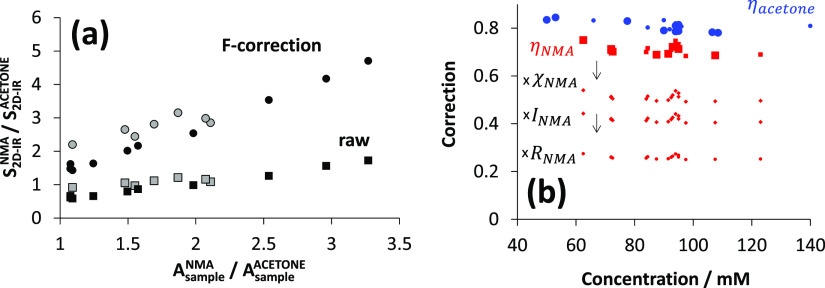
(a) 2D bleach signal and absorption ratios for NMA and acetone signals and IR absorption are well correlated. Better correlation is observed for measurements 8–17 (black points) compared with measurements 1–8 (gray points). (b) Sample-dependent pump absorption correction factors η (large data points, blue acetone, and red NMA) and the effect of three further correction factors for NMA (small data points).

For correcting the concentration ratio measurements 1–17, eq 13 pump absorption correction factors  were computed using the measured values of sample absorbance (*A*_sample_) and total absorbance (*A*_total_), as defined in Figure 3a. The values obtained are shown in Figure 6b.  is plotted as a function of the concentration. In general, the required NMA correction is stronger than that for acetone as the background and sample absorption values are higher for NMA.

### FT-IR/2D-IR Transition Dipole and Concentration Ratios

The 17 random sample acetone and NMA 2D-IR signal values of Figure 4e show substantial variability. The most likely source of this are pump intensity and pathlength variations. Calculating ratios of the NMA and acetone signals for each 2D-IR spectrum eliminates much of this variation. Sample-to-sample ratios of NMA and Acetone 2D signal are well correlated with their corresponding ratios of IR absorbance, as shown in Figure 6a. The noisier measurements 1–7 are made distinguishable from measurements 8–17 by color (gray vs black). The effect of the application of the correction factors is also shown in Figure 6a—the corrected NMA signal size is increased relative to acetone. The sample-to-sample variation of the pump absorption correction factor η for acetone and NMA is shown in Figure 6b. Successive application of the other correction terms is also shown for NMA.

**Figure 7:**
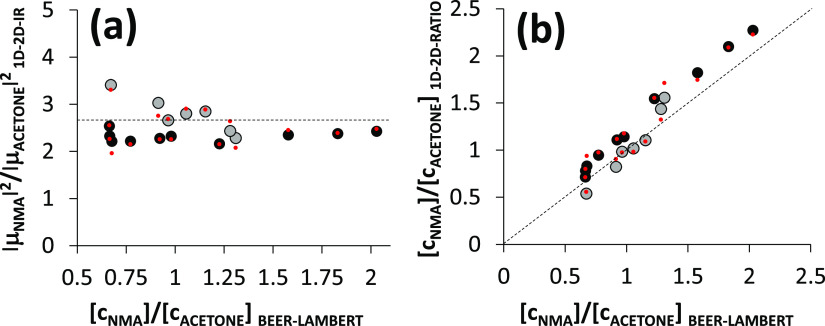
FT-IR/2D-IR transition dipole square ratios and concentration ratios calculated from sample 1–17 2D-IR signals (corrected) and IR absorbances compared with the concentration ratios of the same samples calculated from the Beer–Lambert law. The dashed line in (a) is the value of the squared transition dipole ratio calculated from the Beer–Lambert law. The dashed line in (b) is the line of perfect match with Beer–Lambert law-determined concentration ratios. Measurements 1–7 are shown in gray. Measurements 8–17 are shown in black. The red points are derived from IR-pump-probe measurements using the modified ratio calculations of eq 15.

Figure 7a shows the FT-IR/2D-IR squared transition dipole ratio calculated from the IR and 2D-IR data using the correction factors, FT-IR line widths, and eq 5. Each measurement is plotted against the corresponding Beer–Lambert determined concentration ratio. The average value obtained from the 17 FT-IR/2D-IR ratio measurements is 2.5 ± 12% (standard deviation). The dashed line shows the NMA/acetone carbonyl stretch squared transition dipole ratio calculated separately from the Beer–Lambert law and eq 3—found to be 2.72 ± 2% (combined uncertainty). In FT-IR/2D-IR ratio measurements 8–17, the concentration dependence is perfectly eliminated, resulting in very little spread from measurement to measurement. The spread in values for measurements 1–7 is thought to be caused by the pump laser spectral intensity drift. The lower spread of 8–17 is indicative of better laser stability. Some of the systematic offset may be due to the pump intensity distribution being different to that used for correction (recorded prior to measurement 1) or due to the atmospheric water absorption lines reducing the accuracy of the intensity correction (Figure 3b). An additional 10% uncertainty arises from the signal relaxation correction estimate (Table 1).

A plot of the calculated FT-IR/2D-IR concentration ratios (eq 6) versus the Beer–Lambert determined concentration ratios, as shown in Figure 7b, shows that eq 6 and the Table 1 correction factors predict concentration ratios to within 20% of the correct value, providing a reasonable validation of the FT-IR/2D-IR concentration ratio approach and the correction factors. Measurements 1–7 and 8–17 mostly straddle either side of the correct result. Correlated to the laser intensity instability, the systematic inaccuracies follow a similar pattern to that observed for the transition dipole square determination—measurements 1–7 have a larger variance, while measurements 8–17 do not, instead having a systematic error.

### Transition Dipole and Concentration Ratios Via IR-Pump–Probe Measurements

The expression for a pump–probe bleach-SE signal is almost identical to the 2D-IR expression (eq 4), but as a 1D technique, the signal scales inverse linearly with homogeneous line width Δω [2D-IR signal (eq 4) is inverse-quadratic]. As a consequence, the FT-IR/2D-IR transition dipole and concentrations ratios (eqs 5 and 6) for pump–probe spectroscopy become
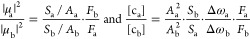
15

It is built-in to every three-pulse pump–probe 2D-IR measurement that for every set of *t*_1_ interferograms acquired to determine a 2D-IR spectrum, the *t*_1_ = 0 value is a pump–probe spectrum. To confirm the transition dipole and concentration ratios of eq 15, bleach-SE amplitudes were extracted from the *t*_1_ = 0 pump–probe spectra for 2D-IR measurements 1–17 and used with their corresponding IR absorption values, correction factors, and IR-measured line widths. These are plotted in Figure 7 as small red data points and almost exactly match the 2D-IR ratios, further demonstrating that the relatively simple theoretical framework of eqs 3–6 and 15 captures the most important effects for determining signal sizes and calculating transition dipole and concentration ratios. As the IR line widths were this time used to calculate the concentration ratio (whereas for 2D-IR, they are not needed), the match also confirms the assumption that the acetone and NMA 2D-IR line shapes are approximately homogeneous—further evidence that the systematic errors in Figure 6 are from the pump spectral intensity and possibly from relaxation corrections, as opposed to errors in the measure of the line shape.

## Conclusions

In this paper, a framework for calculating the concentration ratio of two analyte species from 2D-IR or IR-pump-probe diagonal bleach signals and IR absorption strengths, without knowledge of either species’ molar absorption coefficients or concentrations is explored and experimentally tested using aqueous solutions of two carbonyl containing compounds at random concentrations between 50 and 200 mM and at concentration ratios between 0.5 and 2. The chosen compounds were straightforward to independently analyze using conventional spectrophotometric concentration analysis (based on the Beer–Lambert law). Achieving quantitative accuracy required the determination of correction factors to account for pump absorption, sample relaxation, laser spectral intensity, and bleach-SE/ESA signal overlap. Without these, the NMA/acetone concentration ratio would have been incorrect by a factor of 2.5×. Agreement of the sample’s FT-IR/2D-IR concentration ratios to within 20% of the Beer–Lambert ratios was obtained, validating of the approach. The differences were likely due to variation in laser spectral intensity over some of the measurements, and to systematic errors in the characterization of the laser spectral intensity and sample relaxation dynamics. The calculated uncertainty associated with each correction factor for both samples combined was 20%—comparable to the observed inaccuracy of the measured concentration and transition dipole square ratios. This may be reduced in future through better characterization of the pump spectrum and the use of 2D-IR spectrometers based on intrinsically more stable IR laser technology.^26,32,33^

In addition to concentration determination, the 2D-IR framework for deducing transition dipole ratios published previously^8,9,11^ was revisited, re-cast with the additional correction factor *F* and along with the concentration ratio, modified to include pump–probe spectroscopy. For the samples studied, it was shown that either 2D-IR spectroscopy or IR pump–probe spectroscopy was equally good in combination with FT-IR for obtaining concentration ratios and transition dipole ratios, though the three techniques taken together probably form the best route to robust analysis. For congested (quasi-continuous absorption) IR spectra, the band fitting required for quantification of IR absorption strengths may be considerably easier when a 2D-IR spectrum is available to provide fitting constraints. This is even more so for pump–probe spectroscopy. Pump–probe spectroscopy provides poor separation of the diagonal bleach-SE signals of one sample component with overlapping ESA bands of other components. In 2D-IR spectroscopy, the signals are spread into two spectral dimensions, giving a better separation of overlapping spectral components. Neither FT-IR nor pump–probe spectroscopy readily provide information about the line shape origins of the bands studied, making corrections for inhomogeneous broadening and anharmonicity more difficult to determine. A reason to establish such confidence limits for using pump–probe IR spectroscopy in concentration and transition dipole analysis is that pump–probe spectra can often be acquired far more rapidly and with intrinsically better signal-to-noise than 2D-IR spectra. This may make the pump–probe approach particularly suited to time-limited applications where the species to be characterized are weak in absorption and/or highly transient after preparation.

We have discussed the measurements here as being ratiometric for any two species a and b. The sample could also comprise more than two unknown species, with each then determined pairwise with respect to the others. It also follows that the use of a calibrant molecule added to the sample as species “a,” with known concentration and known transition dipole moment, allows the determination of the absolute concentration and transition dipole moment of species “b”. Previous 2D-IR analyses for computing absolute transition dipole moments with a calibrant^9,10,24,25^ mention some of the possible sources of error (correction factors) tackled explicitly in this work but avoid their determination by use of the fact that if the calibrant has a similar homogeneous and inhomogeneous line width, anharmonicity, and vibrational relaxation to the species analyzed, the correction factors cancel, simplifying the calculation of absolute transition dipole moments (or concentrations). These studies also neglect pump absorption, which is shown here to matter if the optical densities of a and b are >0.1 and differ from one another by more than 0.1. For the NMA/acetone ratio computed in D_2_O, without the corrections described in the manuscript explicitly accounted for, the concentration and transition moment square ratios are 2.5× too large and too small respectively—a substantial difference. Going systematically through the corrections described in this manuscript will therefore improve the confidence/accuracy of ratiometric determinations when using calibrants.

The measurements presented in this paper may be useful in chemical applications where IR spectroscopy provides useful spectra–structure correlations but where concentration information and absorption coefficients for the spectral components cannot be readily obtained. The approach may be particularly relevant where alternative methods such as nuclear magnetic resonance, gravimetric approaches, chromatography, and mass spectrometry are difficult to apply or ambiguous in results. This could include the study of the structure and composition of catalysts such as zeolites, geological samples, and rare materials containing poorly characterized impurities. FT-IR/2D-IR ratio methods might also be useful for exploring the stoichiometry of reactants and products formed from complex solution or solid phase chemical processes. 2D-IR spectroscopy is predominantly applied in the condensed phase; however, the study of gas phase samples is also becoming more common,^34−36^ with FT-IR/2D-IR ratio measurements potentially applicable. Recent advances in the quality and scope of excited state 2D-IR (“transient TR-2D-IR”) spectroscopy^37^ may also provide a route to photoproduct concentration and transition strength determinations. In a TR-2D-IR experiment, the required IR absorption data—the excited-state photoproduct IR absorption spectrum, or “TR-IR” spectrum, are collected as part of the measurement of the TR-2D-IR spectrum and therefore are available in every TR-2D-IR data set for FT-IR/2D-IR ratio analysis.

## Supporting Information Available

2D-IR experimental details; determination of anharmonicity, laser intensity, and relaxation correction factors; further justification of the units of 2D-IR signal; and a projection of the 2D-IR signal in OD/(cm^–1^)^1/2^ units to a pump–probe spectrum in OD units (PDF)
