# Supplementary material for: Spectrophotometric Concentration Analysis Without Molar Absorption Coefficients by Two-Dimensional-Infrared and Fourier Transform Infrared Spectroscopy
Source: Anal Chem. 2022 Dec 14;94(51):17988–99. doi: 10.1021/acs.analchem.2c04287 (PMC9798376; doi:10.1021/acs.analchem.2c04287)
Supplement: Supplementary file 1 — ac2c04287_si_001.pdf [file ac2c04287_si_001.pdf]

Supporting information

Spectrophotometric concentration analysis without molar absorption coefficients by 2D-IR and FT-IR spectroscopy

Paul M. Donaldson*

Central Laser Facility, RCaH, STFC Rutherford Appleton Laboratory, Harwell Science and Innovation Campus, Didcot, OX11 0QX, UK

paul.donaldson@stfc.ac.uk

Table of contents

1. 2D-IR spectroscopy measurements. Page S1

2. Determination of the correction factors F from Eq. 7 of main text. Page S2

2.1 The FT-IR / 2D-IR ratio signal corrections: laser intensity I(ω)

2.2 Signal corrections: anharmonicity

2.3 Signal corrections: Relaxation term R(T_1,τ_{rot})

3. A justification for the mOD / √cm^{-1} units of 2D-IR spectra. Page S4

1. 2D-IR spectroscopy measurements

2D-IR spectroscopy: The Ultra B 2D-IR spectroscopy system at the Central Laser Facility, UK was used for the 2D-IR measurements. A three-stage Ti:Sapphire amplifier system (Coherent Legend Elite Duo, 20W, 50 fs, 10 kHz) was used to drive two Optical Parametric Amplifiers (Light Conversion Topas Prime) with difference frequency generation in $AgGaS_2$ to generate IR light at a centre wavelength of 1700 cm^{-1} and a FWHM of roughly 170 cm^{-1} (pump) and 300 cm^{-1} (probe). The pulse energies at OPA output were 1.6 and 1.2 μJ respectively. The pump was routed through an IR pulse shaper (Phasetech Spectroscopy) and the probe routed over a computerised optical delay line. Both beams were parallel polarised and focussed / overlapped to a spot of ~90 μm FWHM at the sample position using a 10 cm focal length off-axis parabolic mirror. The transmitted probe beam was collimated and spectrally dispersed onto a 128 element MCT array detector (Infrared Associates). A simple optimisation of second-harmonic generation of the pump from $AgGaS_2$ was used to roughly determine dispersion compensation settings for the pulse-shaped pump. The instrument response determined by the spectrally dispersed pump-probe optical Kerr effect was 200 fs around the centre-wavelength (1700 cm^{-1}) and around 260 fs at 1620 cm^{-1}. These high values were due to both the crude pulse shaper dispersion compensation and several CaF2 optical elements in the probe beam path.

4-frame phase cycling and t_1 coherence time steps of 18 fs to a maximum of 3 ps were used to record three-pulse pump-probe 2D-IR interferograms, giving a pump spectral resolution of ~5.5 cm^{-1}. The probe spectral resolution was ~3.5 cm^{-1}. Signal averages of 15 minutes were used for all

measurements. This relatively long acquisition time was necessary due to sub-optimal operation of the Ti:Sapphire amplifier used. Some of the probe light was used as a reference and dispersed on a 32 element MCT detector. Reference correlation[1] improved the quality of chopped pump-probe measurements substantially, however the improvements were found to be less significant for the Fourier transformed 2D-IR spectra, presumably because the 10 kHz pulse-shaper interferogram acquisition rate also removed much of the probe noise.

2. Determination of the correction factors F from Eq. 7 of main text

2.1 The FT-IR / 2D-IR ratio signal corrections: laser intensity I(ω)

Laser spectral intensity determination and stability were one of the biggest sources of error for the concentration ratio measurements. A pump laser spectrum measurement is shown in Figure 3(b) (main text). The 2D-IR experimental setup did not have a particularly efficient N_2 purge, and the gaseous water absorption lines are noticeable in the spectrum. The systematic accuracy of the determination of the laser intensity at the carbonyl absorption frequencies was therefore reduced, and estimated to be ~10%. The correction terms were defined relative to the laser intensity at the acetone carbonyl frequency and therefore 1 for acetone and 0.82 for NMA. The laser spectral intensity was measured prior to beginning the measurement series 1-7. Figures 6 and 7 (main text) show that it is likely that the Ti:Sapphire amplifier and OPA performance was unstable during measurements 1-7, resulting in the pump intensity spectrum drifting, and so introducing a random error. Under the more stable operation during measurements 8-17, the random error became a systematic error. Although some of this systematic error may be from a fixed change in pump laser spectrum or from the water absorption lines in the pump spectrum, it is also possible that the grating spectrograph used to measure the pump intensity spectrum in Figure 3(b) (main text) may not accurately reproduce the pump intensity distribution. Possible contributing effects could be wavelength dependence in the grating diffraction efficiency and the imaging of the laser spectrum onto the array detector. A better purge, more stable laser and determination of the pump spectrum by interferometry (using the pulse shaper) would give more confidence in the pump spectral intensity measurement.

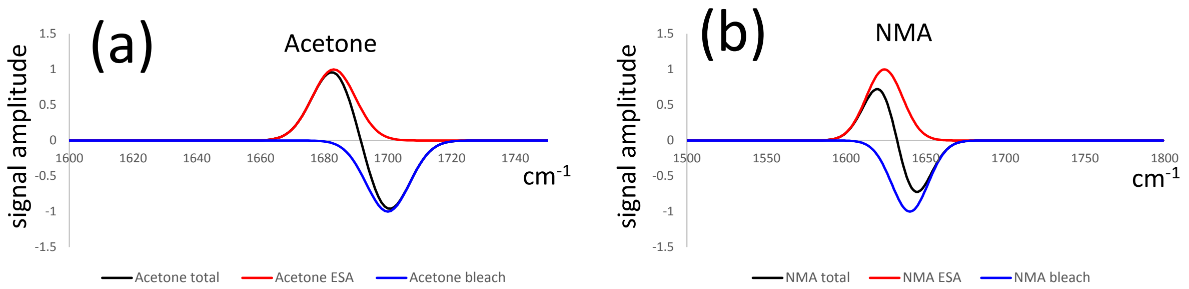

Figure S1. The calculation of the anharmonicity correction factor using Gaussian peaks for negative bleach-SE and positive excited state absorption signals of (a) Acetone and (b) NMA by using the widths and anharmonic shift as described in the text.

2.2 Signal corrections: anharmonicity

The acetone carbonyl stretch overtone band frequency was determined by FT-IR to be at 3378 cm^{-1} and the fundamental at 1697.5 cm^{-1}. The anharmonicity Δ_{an} ($2\omega_{01}$-ω_{02}) is therefore ~17 cm^{-1}. The acetone carbonyl stretch band's FWHM (FT-IR) is 16 cm^{-1}. On simulating the bleach-SE and ESA using a pair of Gaussian peaks and these parameters, no interference between bleach and ESA is observed ($\chi(\Delta_{an},\Delta\omega)$ =1, Figure S1(a)). Due to ω_{02} overlap with the NH stretch band, the anharmonicity of the

carbonyl stretch of NMA is harder to observe by FT-IR, but known from the literature[2] to be ~16 cm^{-1}. The FWHM of the NMA carbonyl stretch is 27 cm^{-1}. This causes substantial overlap of the bleach-SE and ESA signals (Figure S1(b)). A 1D probe cut along the peak centre of an experimental 2D-IR spectrum was found to be well described by Gaussian lineshape functions, and $\chi(\Delta_{an},\Delta\omega)$ was calculated to be 0.72 for NMA. Assuming that the FT-IR measured linewidth and literature anharmonicity are accurate to ±1 cm^{-1} gives an uncertainty of 4%.

2.3 Signal corrections: Relaxation term $R(T_1,\tau_{rot})$

The bleach-SE signal decay as a function of t_2 for the NMA and acetone carbonyl bands were measured using pump-probe spectroscopy (Figure S2). NMA signal relaxation was observed to be bi-exponential, with time constants of ~0.25 and ~0.95 ps at weightings of 0.6 and 0.4 respectively. These values are in good agreement with those reported by De Camp et al.[3] The acetone bleach-SE decay fit well to a single exponential with a 1.8 ps decay time. Fits to the NMA experimental data are shown in Figure S2(a). The fast component of the NMA relaxation is similar in timescale to the instrument response, and it was necessary to account for this using a simple single frequency model of the bleach signal based on convoluting the laser pulse envelope *I* for the pump with the sample relaxation term, and taking a second convolution of this response with the probe field:

$$S(t_2) \propto \int E_{probe}(t-t_2).[\int I_{pump}(t-t_1).\left(A_1 e^{-{t_1}/{T_{rel\,1}}} + A_2 e^{-{t_1}/{T_{rel\,2}}}\right)dt_1]\, dt \qquad \text{(S1)}$$

Eq. S1 was integrated numerically using Gaussian shaped laser pulses. 2D-IR measurements 1-17 were recorded at an experimental t_2 delay of 300 fs. In the experiment, t_2=0 was defined near the peak of the acetone bleach response. To achieve the fit to the experimental NMA data in Figure S2 (a), pulse-widths of 260 fs and 130 fs were used for the pump and probe pulse intensities, and a relative probe delay (time zero correction) offset of -75 fs. As a consequence of the broad pulses and chirp, the NMA signal size peaks ~250 fs later than the experimental time zero. The acetone bleach also fit well to Eq. S1, with a single exponential (time constant 1.8 ps), a pump pulse duration of <200 fs and a relative probe delay offset of +50 fs (Figure S2 (b)). The abrupt change leading to time zero is weak Kerr response (often referred to as 'coherent artefact') and the negative time signal is perturbed free induction decay. Both are ignored in the fitting.

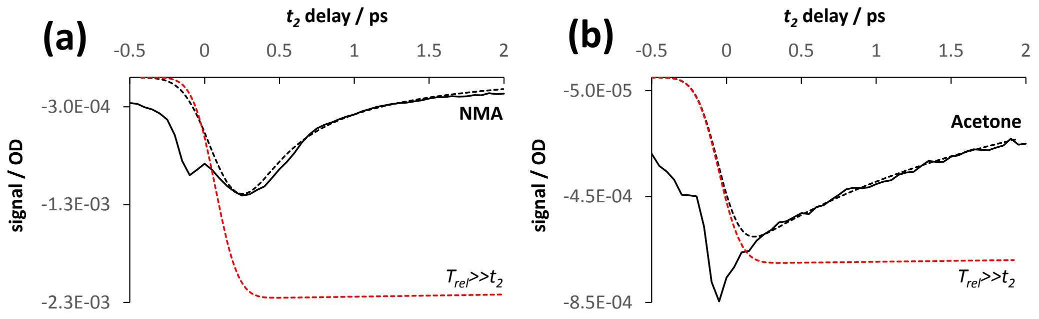

Figure S2. Population and reorientational relaxation reduces the amplitude of the peak NMA (a) and the peak Acetone (b) bleach signals (measured at ~ 1620 and 1700 cm^{-1} probe frequency). Experimental bleach decays (solid lines) are shown with fits (dashed) obtained using convolutions of the laser pulse with the exponential sample relaxation terms. A single exponential decay describes the acetone bleach decay and a bi-exponential decay describes NMA. Calculated in red is the bleach amplitude obtained by making the relaxation terms large.

The fitting of the laser pulse parameters in Eq. S1 could potentially be erroneous or biased. To estimate the corrections to the 2D-IR signals and get an idea of the potential uncertainty, three scenarios of increasing accuracy were therefore considered. These are shown in Table S1. The first is simply to disregard the complexities of the instrument response, chirp and pulse timings by assuming delta function laser pulses, and use the bi and mono-exponential decay amplitudes obtained for NMA and acetone at the experimentally estimated waiting time t_2=300 fs as the correction factor. The second scenario incorporates the instrument response effects of Eq. S1 but assumes an ideal case of transform-limited 100 fs pump and probe pulses. The correction factors are calculated as the ratio of the calculated NMA or acetone signal amplitude at t_2=300 fs relative to the signal amplitude obtained when the lifetime of the bleach is instead very large (in other words, the ideal case of setting the relaxation terms to be very large for the fitted curves, red lines, Figure S2). The third (more accurate) calculation again uses the instrument response calculation of Eq. S1, but also incorporates the chirped pulse parameters obtained from the fits in Figure S2. The three calculations give similar results. The chirped pulse corrections of Table S1 (case 3) were used for the correction factors with an uncertainty taken to be ±10% - the spread of the value of the ratio of the corrections in Table S1.

**Correction (t_2=300fs)**	**1. Kinetics only (delta pulses)**	**2. 100fs laser pulses**	**3. Chirped pulses**
$R_{acetone}$	0.85	0.85	0.81
R_{NMA}	0.49	0.47	0.50
$R_{NMA}/R_{acetone}$	0.58	0.55	0.62

Table S1. Correction factors for NMA and acetone relaxation $R(T_1,\tau_{rot})$ at three levels of approximation

3. A justification for the mOD / √cm^{-1} units of 2D-IR spectra

This section expands the discussion around the units of 2D-IR spectra. Owing to the heterodyne process of a third order signal measurement being naturally ratiometric, the required t_1 interferograms for 2D-IR spectroscopy (or the transformed data) are often calculated in units of optical density, regardless of whether the 2D-IR experiment is conducted in the three pulse pump-probe or four-pulse boxcars geometry. As mentioned in the main text, heterodyning and reference normalisation of the third order signal (whether achieved with the probe, a separate local oscillator or separate reference measurement) eliminates any dependence of 2D-IR signal on the spectral resolution used for determining the ω_3 axis. 2D-IR interferograms $S(t_n,\omega_3)$, in absorbance units, will comprise a set $\{S_{t_n}\}$ of M x t_1 points spaced by time interval Δt for a given probe detector pixel ω_3. Data treatment prior to Fourier Transform usually includes zero padding, which increases the number of interferogram points to $2M=N$.[4] Window functions are sometimes applied, which may reduce 2D-IR signal amplitudes, but used appropriately, this effect should be small.

The standard DFT algorithm often used for processing 2D-IR data and supplied with many common programming packages is the Fast Fourier Transform (FFT), which calculates the following:[5]

$$DFT[\{S_{t_n}\}] = \{S_{\omega_k}\} = \{\sum_{n=0}^{N-1} S_{t_n} e^{-i2\pi kn/N}\} \quad \text{(S2)}$$

Eq. S2 transforms a real-valued interferogram to the frequency domain using the complex exponential basis set. In this basis set, the real part of the FFT are sums of complex exponentials with opposite exponent sign. This explains why duplicate 2D-IR spectra with positive and negative frequencies are observed in the real output of Eq. S2. In the following arguments, ω is in units of rad s^{-1}.

The real part of Eq. 17 will not give a constant 2D-IR spectral amplitude under varying N and Δt. A normalisation factor for 2D-IR spectroscopy must therefore be chosen to ensure that the step size and spectral resolution can be changed without causing any change in 2D-IR spectrum signal amplitude. To proceed, first we recognise that to approximate a continuous FT, the standard FFT algorithm of Eq. S2 requires multiplication by Δt:

$$FT[S(t)] = \int S(t)e^{-i\omega t}dt \approx \Delta t.DFT\left[\{S_{t_n}\}\right] = \Delta t.\{S_{\omega_k}\} \qquad \text{(S3)}$$

Parseval's theorem must be satisfied for the correct normalisation. The continuous and discrete forms are:

$$\int |S(t)|^2 dt = \frac{1}{2\pi}\int |S(\omega)|^2 d\omega \quad \leftrightarrow \quad \sum_{n=0}^{N-1} {S_{t_n}}^2 \Delta t = \frac{1}{2\pi}\sum_{k=0}^{N-1} (S_{\omega_k}\Delta t)^2 \Delta\omega \qquad \text{(S4)}$$

The frequency spacing $\Delta\omega$ is $2\pi/N\Delta t$. Therefore, for FFT data from Eq. S2, the discrete version of Parseval's theorem can be rearranged to give:

$$\sum_{n=0}^{N-1} {S_{t_n}}^2 \Delta t = \sum_{k=0}^{N-1} \left(\sqrt{\frac{\Delta t}{N}} S_{\omega_k}\right)^2$$

(S5)

It follows that the amplitudes of the real part of Eq. S2 are properly normalised, and thus independent of step size and number of samples when multiplied by $\sqrt{\Delta t/N}$. The units of the FFT of a set of 2D-IR interferograms in this normalisation scheme are therefore absorbance $Hz^{-1/2}$. Under this set of units, the result of FFT is a *spectral amplitude,* as opposed to the more common measure of signal intensity: the *power spectrum*[5] (which has units of Amplitude squared / Hz). The power spectrum is the transform of the signal squared, and therefore inappropriate for 2D-IR spectroscopy. These unit definitions come entirely from the discrete nature of the FFT along the ω_1 pump axis.

If the units of time for the 2D-IR interferogram signal $S_{2D-IR}(t_n,\omega_3)$ t_1 points are femtoseconds, then the units of normalised frequency domain signal $S_{2D-IR}(\omega_1,\omega_3)$ are absorbance. $\sqrt{fs}$. We may convert this to absorbance / $\sqrt{cm^{-1}}$ through multiplication by $\sqrt{c}$, with c the speed of light in cm fs^{-1} (equal to 3 x 10^{-5} cm fs^{-1}). Two other correction factors are needed - the 2D-IR spectral amplitude appears at positive and negative frequencies in the real part of the FFT. Each component carries half the total spectral amplitude (the factor 2 in Eq. 14 of the main text, reproduced below). In the conventional approaches of removing backgrounds with shot-to-shot phase cycling, calculating signals from a sequence of phase cycled measurements increases the apparent signal size relative to 'on-off' optical chopping by the number of phases introduced, n_ϕ .[6] Collecting all these factors, with the timestep size in femtoseconds we arrive at the correction to the 2D-IR spectrum introduced earlier in proper units of absorbance / $\sqrt{cm^{-1}}$ (Eq. 14, main text):

$$S_{2D-IR}(\omega_1,\omega_3)\left(\frac{OD}{\sqrt{cm^{-1}}}\right) = Re\left(FFT\left[S(t_n,\omega_3)\right]\right).\sqrt{\frac{\Delta t}{N}}.\sqrt{3\times 10^{-5}}.2.\frac{1}{n_\varphi} \quad \text{(Eq. 14, main text)}$$

Optical chopping or other variations of signal de-modulation can be accounted for. For example, a 3-pulse pump-probe experiment using 2-state chopping only would use n_ϕ =1. A fast-scanned interferometer,[7] with no chopping or phase cycling (n_ϕ=1) measures signal every laser shot, and so the total signal would be scaled correctly with an additional factor of ½ in Eq. 14 to account for the signal being double the chopped case.

The data in this paper was collected using n_φ = 4 (four-frame phase cycling), a step size of 18 fs, M=167 time steps (final t_1 value = 3006 fs) and N = 2x168 = 336 points (zero-padding). Using these parameters, 2D-IR spectra from the real part of the FFT of the interferograms calculated in absorbance units for the acetone 2D-IR measurement in Figure 3 (main text) had un-normalised peak bleach signal amplitudes of order ~3 x 10^{-2} (unscaled Eq. 17 FFT output), split into two signals at positive and negative frequencies in the real part of the FFT output. Using Eq. 14 (main text), in absorbance units of milliOD (mOD), the acetone peak bleach 2D-IR amplitude is 2 x 10^{-2} mOD / $\sqrt{cm^{-1}}$. The pump-probe acetone bleach signal amplitude was 0.45 mOD (chopped, two-state measurement). How does this relate to the 2D-IR amplitude? Figure S3 shows that a pump-probe spectrum calculated from a 2D-IR spectrum (normalised according to Eq. 14) by summing (projecting) the 2D-IR spectrum along the pump axis, matches *exactly* with the chopped pump-probe spectrum calculated from the 2D-IR interferogram at t_1=0 when the projected 2D-IR spectrum is multiplied by twice the square root of the zero-padded pump spectral resolution ($1/N\Delta tc$), which in the present experiments is $2\sqrt{5.5\ cm^{-1}}$. Examination of the discrete projection slice theorem in a future work might reveal why this particular factor connects the projected 2D-IR spectrum scaled according to Eq. 14 to the pump probe spectrum.

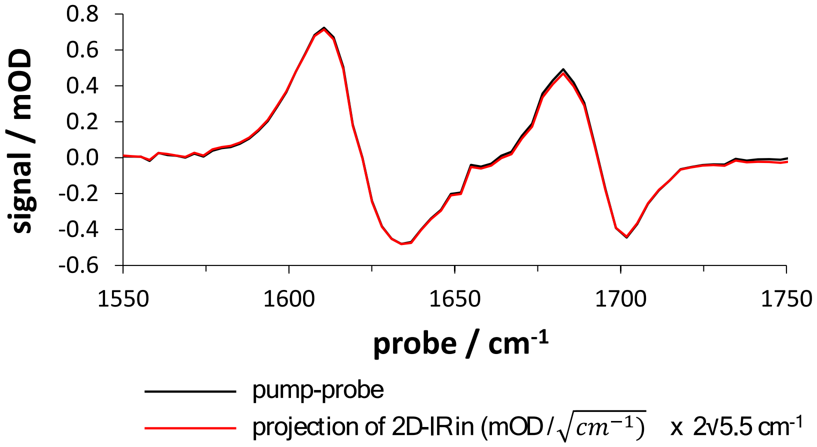

Figure S3. The pump-axis projection of a 2D-IR spectrum plotted in amplitude units of mOD / $\sqrt{cm^{-1}}$ (Eq. 14) multiplied by twice the square root of the pump spectral resolution (red) exactly matches the amplitude of the chopped pump-probe spectrum (black). Both the pump-probe spectrum and the pump-projected 2D-IR spectrum are from the same 2D-IR spectral data of Figure 3(c), main text.

Supplementary information bibliography

(1) Feng, Y.; Vinogradov, I.; Ge, N.-H. General Noise Suppression Scheme with Reference Detection in Heterodyne Nonlinear Spectroscopy. *Opt. Express* **2017**, *25* (21), 26262. https://doi.org/10.1364/oe.25.026262.

(2) DeFlores, L. P.; Ganim, Z.; Ackley, S. F.; Chung, H. S.; Tokmakoff, A. The Anharmonic Vibrational Potential and Relaxation Pathways of the Amide I and II Modes of N-Methylacetamide. *J. Phys. Chem. B* **2006**, *110* (38), 18973–18980. https://doi.org/10.1021/jp0603334.

(3) DeCamp, M. F.; DeFlores, L.; McCracken, J. M.; Tokmakoff, A.; Kwac, K.; Cho, M. Amide I Vibrational Dynamics of N-Methylacetamide in Polar Solvents: The Role of Electrostatic Interactions. *J. Phys. Chem. B* **2005**, *109* (21), 11016–11026. https://doi.org/10.1021/JP050257P/ASSET/IMAGES/MEDIUM/JP050257PE00013.GIF.

(4) Hamm, P.; Zanni, M. *Concepts and Methods of 2D Infrared Spectroscopy*; Cambridge University Press, 2011; Vol. 9781107000. https://doi.org/10.1017/CBO9780511675935.

(5) Press, W. H.; Teukolsky, S. A.; Vetterling, W. T.; Flannery, B. P. *Numerical Recipes in Fortran 77*, Second

Edi.; Cambridge University Press, 1992.

(6) Bloem, R.; Garrett-Roe, S.; Strzalka, H.; Hamm, P.; Donaldson, P. Enhancing Signal Detection and Completely Eliminating Scattering Using Quasi-Phase-Cycling in 2D IR Experiments. *Opt. Express* **2010**, *18* (26), 27067. https://doi.org/10.1364/oe.18.027067.

(7) Helbing, J.; Hamm, P. Compact Implementation of Fourier Transform Two-Dimensional IR Spectroscopy without Phase Ambiguity. *J. Opt. Soc. Am. B* **2011**, *28* (1), 171. https://doi.org/10.1364/josab.28.000171.
